# Exploring the perspectives of pulmonologists on referrals for pulmonary rehabilitation in India: insights into current practices

**DOI:** 10.1038/s41598-026-38711-4

**Published:** 2026-03-07

**Authors:** Anulucia Augustine, Anup Bhat, Aswini K. Mohapatra, Anne E. Holland, K. Vaishali

**Affiliations:** 1 Department of Physiotherapy, Manipal College of Health Professions, Manipal Academy of Higher Education, Manipal, India; 2 Present Address: Pulmonary Rehabilitation, Wirral Integrated Respiratory Services, Clatterbridge Hospital, Wirral University Teaching Hospital, NHS Trust, Clatterbridge, Wirral, CH63 4JY UK; 3 Department of Respiratory Medicine, Kasturba Medical College, Manipal Academy of Higher Education, Manipal, India; 4 Alfred Health and Monash University, Melbourne, VIC 3004 Australia

**Keywords:** Chronic respiratory diseases, Difficulties, Enablers, Limited resources, Pulmonologists, Pulmonary rehabilitation, Health care, Medical research

## Abstract

Pulmonary rehabilitation (PR) is an evidence-based treatment for chronic respiratory diseases that improves physical and psychosocial health. However, global referral rates remain low, especially in resource-limited settings where unique barriers complicate the referral process. This survey examined the facilitators of and barriers to PR referral among pulmonologists in India. A cross-sectional survey of pulmonologists in India was conducted via a validated questionnaire (content validation index of 0.93) with open- and closed-ended questions on facilitators and barriers to the referral of an individual to a PR. A total of 114 pulmonologists participated in the survey. Among the pulmonologists, facilitators of PR referrals included the availability of a PR centre within the hospital or nearby (*n* = 71, 81%), the availability of trained professionals at the PR centre (*n* = 67, 76%), positive beliefs about PR (*n* = 64, 73%) and major barriers were limited centres offering PR (*n* = 70, 80%), and financial constraints for patients (*n* = 58, 66%). According to the pulmonologists, the factors that facilitated patients’ participation in the PR program were good family support (*n* = 73, 86%), patients’ level of motivation (*n* = 68, 80%), easy accessibility to the rehabilitation centre (*n* = 66, 78%), and the barriers were a lack of awareness of the benefits of the PR (*n* = 59, 69%), and inaccessibility to the rehabilitation program (*n* = 55, 65%). Our survey identified various facilitators and barriers encountered by pulmonologists while referring patients to PRs. These factors can inform the development of context-specific referral pathways to improve PR access in resource-limited settings.

## Introduction

Pulmonary rehabilitation (PR) is an integral and comprehensive intervention used for the management of people with chronic respiratory diseases^1^. In 2016, the Global Burden of Disease reported that India accounted for 32% of global disability-adjusted life years (DALYs) due to chronic respiratory diseases^2.^ The incidence of disabling chronic respiratory conditions is increasing alarmingly in developing countries, leading to increased morbidity, frequent hospital admissions, rising healthcare costs, and reduced quality of life^3^. Robust evidence has demonstrated the importance of PR in alleviating symptoms such as dyspnoea, exercise intolerance, reduced functional capacity, poor self-efficacy, and reduced health-related quality of life^4^. Furthermore, PR has been shown to reduce healthcare costs^5^.

Despite its remarkable benefits, PR is underutilized across the globe^6^. A clinical audit carried out in the United Kingdom reported that only 15% of the total eligible patients with chronic respiratory diseases were referred for PR, and only 10% attended the initial assessment^7^. A survey involving American and European participants revealed that 22% of respondents with chronic respiratory diseases had not heard of PR^8^. Only approximately 2% of Medicare beneficiaries with COPD received PR in the United States of America^9^. Another survey conducted in the United Kingdom reported that only 40% of the participating hospitals ran the PR program^10^. A systematic review employing the theoretical domains framework (TDF) revealed that PR referrals and participation are significantly influenced by the environment (e.g., waiting times, transport issues, and healthcare resources), knowledge (e.g., clinician awareness of referral processes, patient understanding of PR), and beliefs about consequences (e.g., perceived safety, expectations of outcomes)^11^.

There is some evidence of PR utilization and benefits in developing countries; however, disparities exist in access between urban and rural areas^12^. India, the most populous developing country, presents differing facilitators of and barriers to PR utilization. These may include variations in health literacy, insurance coverage, transportation, the density of healthcare providers in urban versus rural areas, the availability of government health programs, funding, and the patient’s familial support system^13^. Interviews with several healthcare professionals, including pulmonologists, revealed that many providers, especially in rural areas, expressed a lack of access to or training for PR, limiting their ability to recommend or conduct such programs^14,15^. We acknowledge that understanding the patient’s perspective is crucial; however, in India, many patients are unaware of PR due to limited awareness and the scarcity of centers offering these programs. This made it difficult to recruit patients directly, as access and awareness remain low. Therefore, our study focused on pulmonologists, as they play a key role in the referral process. We believe that understanding the barriers from the provider’s perspective is an important first step in addressing the broader issue of limited PR utilization, with future research needed to explore patient views directly. Thus, our study aimed to explore the facilitators of and barriers to PR referral in India.

## Methods

This cross-sectional survey was conducted in accordance with the relevant guidelines and regulations among pulmonologists in India from September 2019 to January 2020. Approval was obtained from the Institutional Ethics Committee (IEC: 39/2019), Kasturba Hospital, Manipal, Karnataka, India and the study was registered under the clinical trial registry - India with the number CTRI/2019/03/018125.. A purposive sampling strategy was used to recruit pulmonologists practicing in India, as they are the primary specialists responsible for managing chronic respiratory conditions, including but not limited to COPD. Importantly, in the Indian healthcare context, pulmonologists are the main clinicians responsible for referring patients to PR programs. Their role as key decision-makers in initiating PR referrals made them the most appropriate target group for this study. In India, there are no government-specific or nationally standardized guidelines solely dedicated to PR. However, clinicians and healthcare providers often refer to internationally recognized guidelines, such as the American Thoracic Society/European Respiratory Society (ATS/ERS) guidelines, which are commonly used as references for determining patient eligibility and appropriate referrals to PR programs in India. Contact details were obtained through various respiratory societies and medical associations across the country. To further expand our reach, we also employed snowball sampling to identify additional pulmonologists who met our study criteria. Recruitment was not limited by geographic location, hospital, or practice setting. The participants represented a mix of inpatient and outpatient settings and included those in clinical, academic, and private practice roles.

A content validated questionnaire with a total of 19 open- and close-ended questions was used for the survey. The questionnaire was developed after an extensive literature review on barriers to and facilitators of PR referral and was developed on the basis of the theoretical domain framework (TDF)^16^. The TDF guided the development and analysis of this questionnaire. We focused on the domains deemed a priori most relevant to pulmonologist referrals to PR, including beliefs about capabilities, beliefs about consequences, the environmental context and resources, motivation and goals, knowledge, and skills. While the TDF^16^ encompasses 14 domains that target the cognitive, affective, social and environmental influences on behavior, practical constraints limit the scope of the questionnaire. For example, while ‘Emotion’ can influence decision-making, we prioritized domains related to practical considerations and beliefs about PR outcomes, as these were identified as key areas in previous research. ‘Social influence’ was assessed to a limited extent, and further exploration of peer influence and adherence to guidelines could provide additional insights. The developed questionnaire addressed the following features: demographics, physician-related factors, patient-related factors, inpatient rehabilitation, and home-based rehabilitation. (A detailed questionnaire is provided in the supplementary file). The questionnaire was validated via Lawshe’s technique for content validation^17^. Six subject experts were involved in this study, comprising specialists in the fields of cardiopulmonary physiotherapy (*n* = 4) and pulmonology (*n* = 2). Among them, five experts (three in cardiopulmonary physiotherapy and two in pulmonology) were academicians and researchers based in a developing country, representing healthcare systems with limited resources. The remaining expert, a specialist in cardiopulmonary physiotherapy, was based in a developed country and held an academic and research role. This diverse panel ensured input from both low-resource and high-resource healthcare settings. Preliminary drafts received qualitative comments for a few questions that were accepted, and necessary revisions were made. The final draft of the questionnaire was subsequently validated by the same panellists, after which the content validity ratio (CVR) for each question was calculated. Questions with a CVR greater than 0.78 were retained, and a content validity index (CVI) of 0.93 was obtained in our study, which was well above the determined cut-off of 0.8.

Five independent healthcare professionals piloted the online survey for readability and face validity. Individuals were physiotherapists with special interest in the field of cardiorespiratory physiotherapy. Piloting identified any unanticipated problems and ambiguity concerning instructions and questions. This helped us estimate the average time needed for completion. Minor changes were identified that required modification to enhance the clarity of several questions, and then, the online survey was finalized for distribution (Supplementary file).

Following development, pulmonologists were invited to participate through an anonymous survey distributed via the online platform SurveyMonkey (SurveyMonkey Inc., San Mateo, CA, USA). Survey responses were stored on secure servers located in the United States in compliance with applicable data protection regulations, including the General Data Protection Regulation. The questionnaire details were linked to the participant information sheet, and informed consent was provided via survey platform. Voluntarily opening and clicking the link implied consent. The participants were given two weeks to complete the survey. Follow-up emails were sent in the second and third weeks to the participants, which prompted completion of the survey after the initial email, with the aim of optimizing the response rate.

The sample size was initially calculated on the basis of the prevalence of transportation barriers at 80%, as reported in previously published literature^6^, which identified transportation as the most common barrier. This estimate was determined prior to the publication of more recent studies. Following the review of recent surveys and qualitative studies, transportation-related barriers continue to be among the most prevalent, with recent data indicating prevalence rates ranging from approximately 63% to 94%^18,19^. Given that the original estimate was aligned with the higher end of the current findings, we maintained 80% as a conservative and justifiable prevalence for our sample size calculation. A total of 126 completed responses were required with a significance level of 5% and a margin of error of 7%. To account for an 85% nonresponse rate, the number of invitations was increased to 840 email invitations. Despite reminders, response rates remained insufficient, prompting us to extend invitations by an additional 200. The survey was anonymous, with optional questions aimed at improving response completeness; thus, denominators varied across different results depending on participants’ response choices. Most questions were closed-ended with predefined response options. Some items included an optional “Other, specify” field to capture additional perspectives not covered by the listed options. These responses were reviewed, but they were closely aligned with existing choices and did not generate new ideas; therefore, formal qualitative or saturation analysis was not undertaken. Responses were analysed via SPSS Statistics for Windows, version 16.0 (SPSS Inc., Chicago, IL, USA), to obtain descriptive and frequency analyses of the data.

## Results

**Fig. 1:**
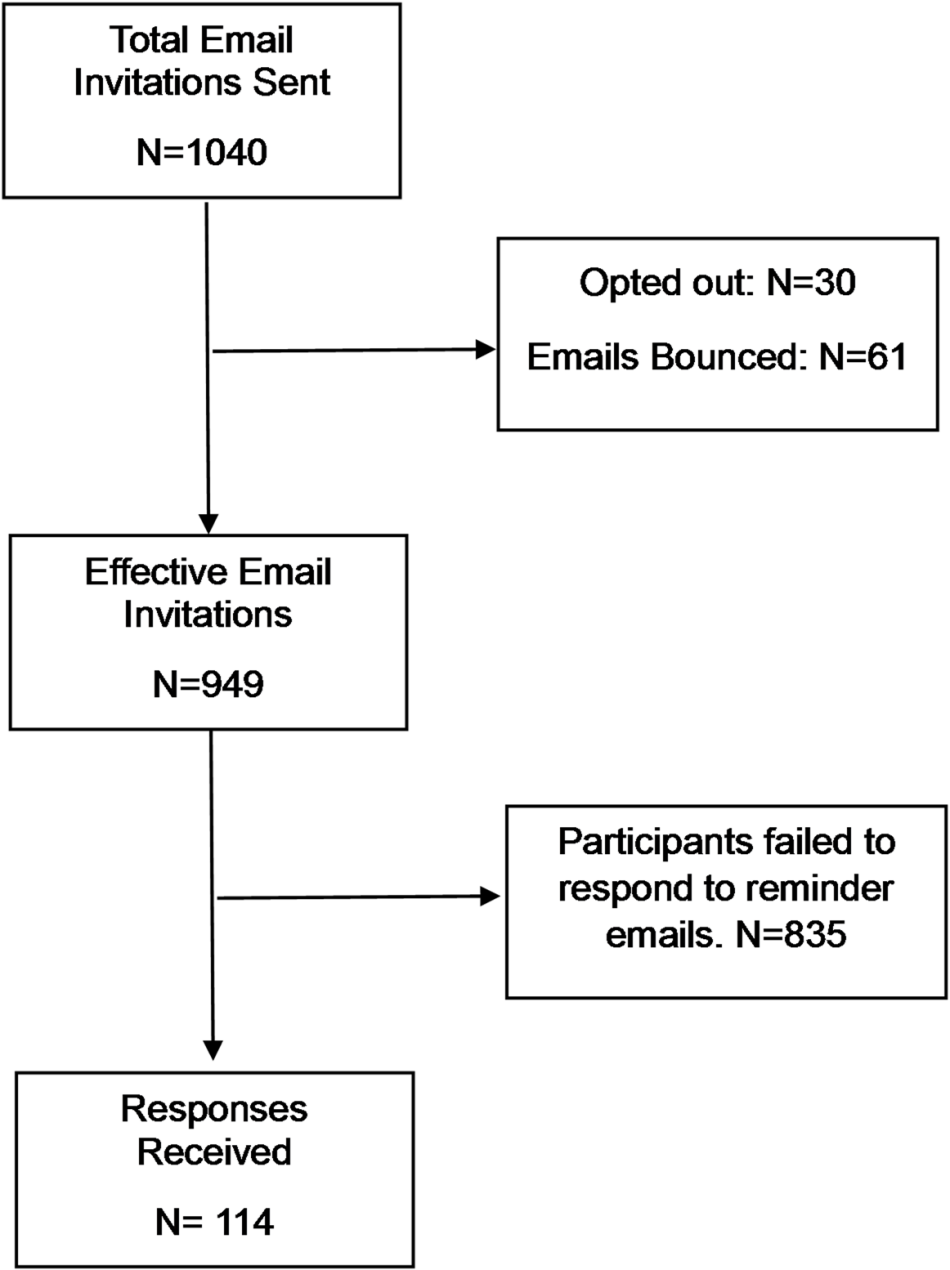
Participants’ flow diagram.

**Fig. 2:**
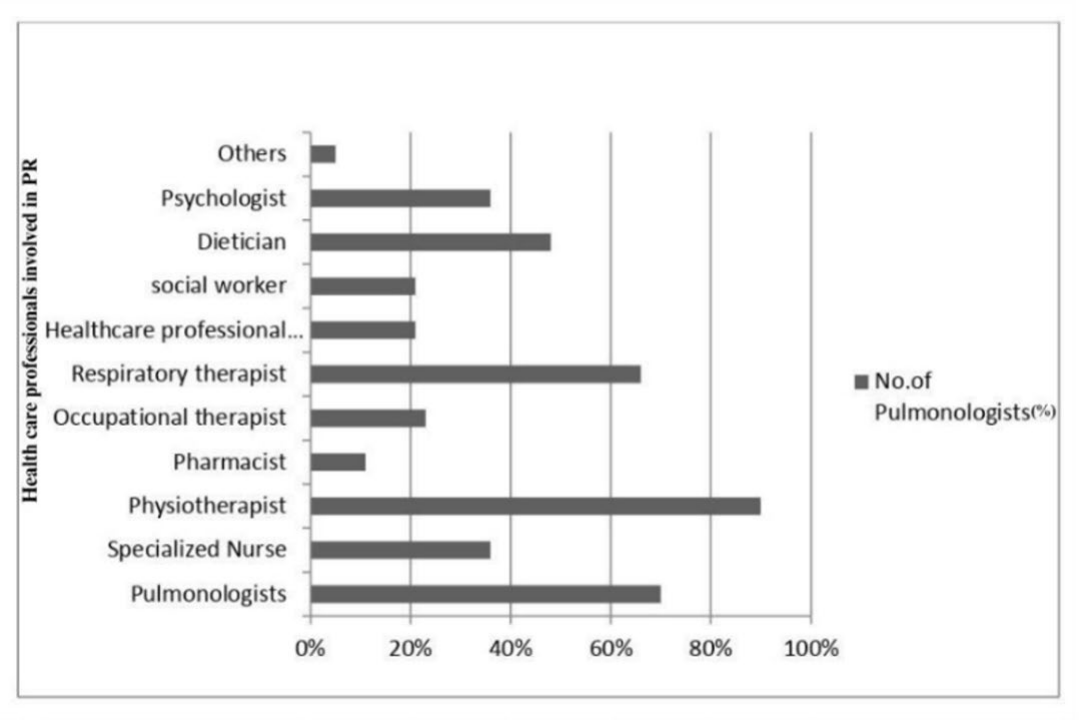
PR team members reported by pulmonologists (N: 114 pulmonologists).

Of the 1,040 pulmonologists invited, 30 opted out, and 61 emails bounced, resulting in 949 effective invitations. A total of 114 pulmonologists responded. The remaining pulmonologists did not respond to any follow-up reminder emails and hence were not contacted further (Fig. 1). The median number of years of experience of participants as clinicians was 16 years (IQR: 6, 20), and the median number of years of experience as academicians was 12 years (IQR: 8, 17.75). The demographic details of the participants are reported in Table 1. Nearly half of all pulmonologists (47%) reported that they were associated with the PR centre, and team members of the PR centre are mentioned in Fig. 2.

**Table 1:** Demographic details.

	N*	Percentage %
1. Educational qualification (*N* = 114)		
MD^a^/DNB^b^ (Pulmonary Medicine)	88	77%
DM^c^ (Pulmonary Medicine)	4	4%
Diploma in Pulmonary Medicine	7	6%
Others like certifications, fellowships.	15	13%
2. Workplace (*N* = 114)		
Academic Research Institution	22	19%
Medical College	64	56%
Private Clinic	25	22%
Corporate hospital	29	25%
Public Hospital/Government Hospital/ Municipal Hospital	10	9%
Charitable/Trust Hospital	11	10%

* Percentages may not add up to 100%, as respondents had the option of choosing more than one workplace.

^a^MD- Doctor of Medicine,

^b^DNB-Diplomate of National Board,

^c^DM- Doctorate of Medicine.

### Pulmonologist factors

**Fig. 3:**
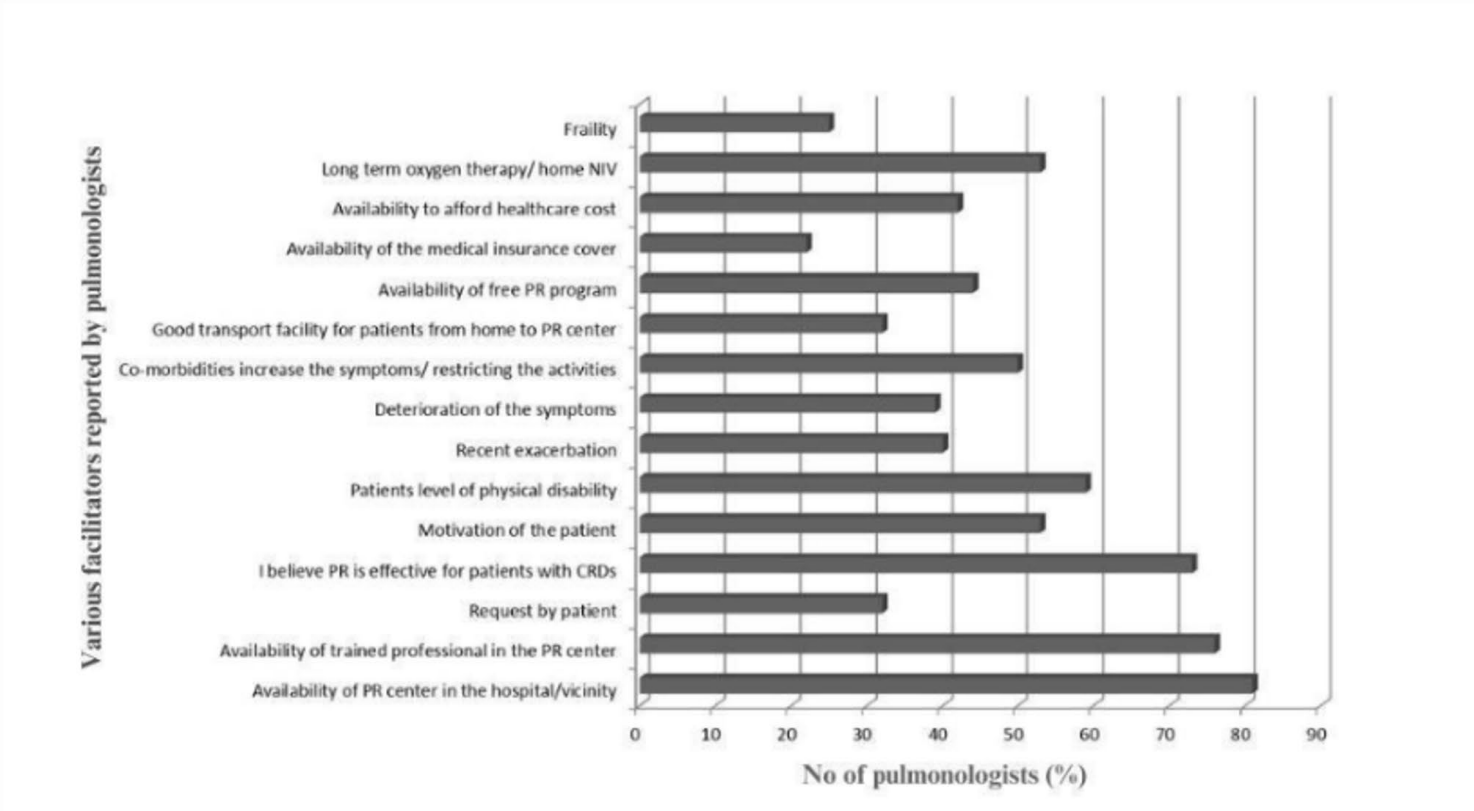
Facilitators of PR referral (N: 114 pulmonologists). Abbreviation: CRD, chronic respiratory disease; NIV, non invasive ventilation; PR, pulmonary rehabilitation.

**Fig. 4:**
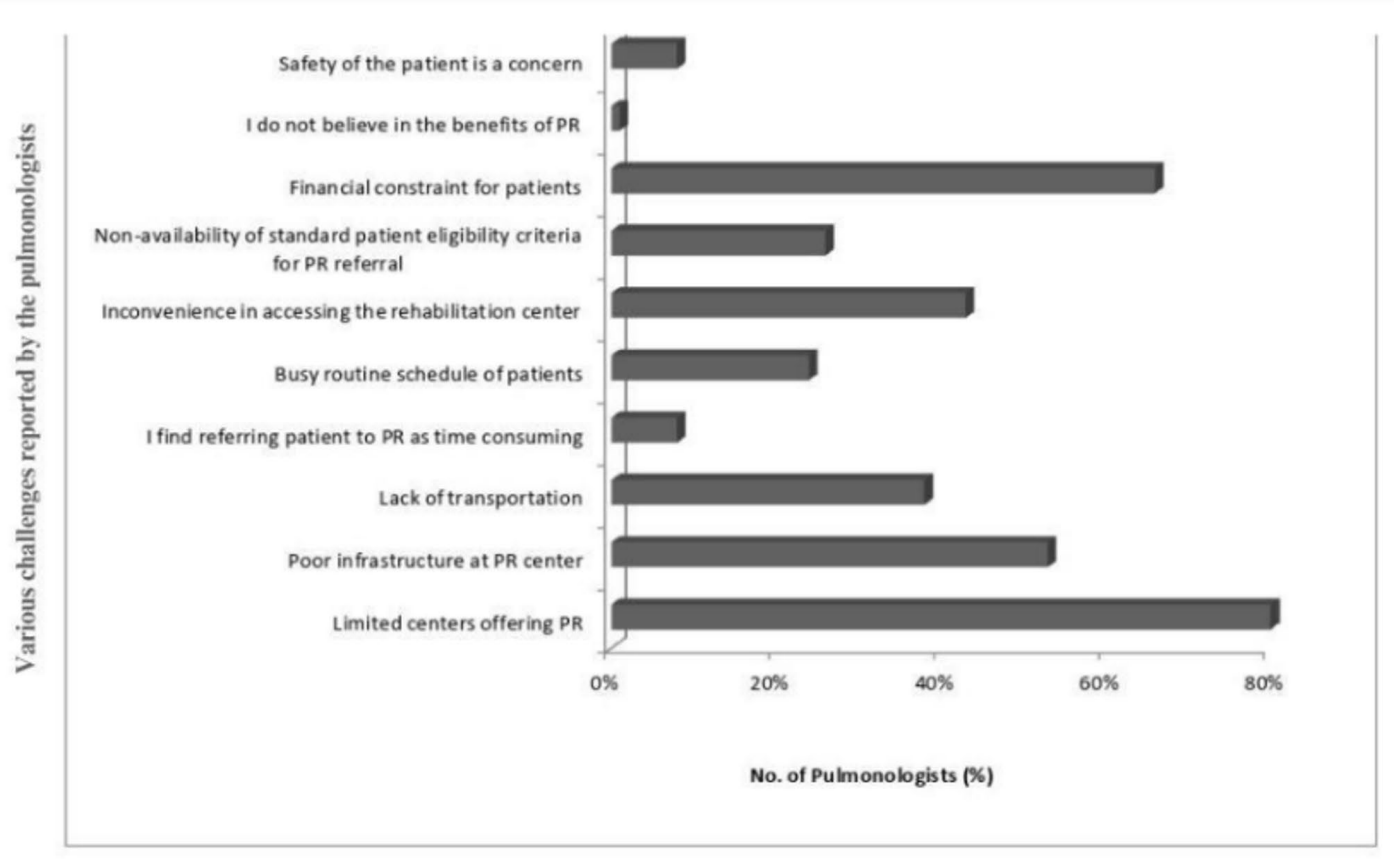
Barriers to PR referral (N: 114 pulmonologists). Abbreviation: PR, pulmonary rehabilitation.

Among the 114 participants, 88 (77%) pulmonologists responded to the questions on facilitators and barriers to referral for PR. Pulmonologists reported that the availability of the PR centre in the hospital/vicinity (*n* = 71, 81%) and the availability of trained professionals (*n* = 67, 76%) were the common facilitators of PR referral, whereas major barriers were the limited number of centres offering PR (*n* = 70, 80%) and financial constraints for patients (*n* = 58, 66%), as depicted in Figs. 3 and 4.

### Pulmonologists’ perceived patient factors

According to the participating pulmonologists (*n* = 85/114, 75%), the factors that would facilitate patients’ participation in the PR program were good family support (*n* = 73, 86%), patients’ level of motivation (*n* = 68, 80%), easy accessibility to the rehabilitation centre (*n* = 66, 78%), positive influence of the pulmonologists (*n* = 65, 76%), positive belief toward rehabilitation (*n* = 63, 74%), ability to afford healthcare costs (*n* = 60, 71%) and good health literacy (*n* = 54, 64%). In addition, pulmonologists reported the common reasons provided by the patients when they refused to participate in the PR program, which were lack of awareness of the benefits of PR (*n* = 59, 69%), inaccessibility to the rehabilitation program (*n* = 55, 65%), lack of financial assistance (*n* = 51, 60%) and fear of exercise causing breathlessness (*n* = 44, 52%).

### Inpatient PR referral

Approximately 53% (*n* = 45/85) of the pulmonologists responded to having a provision for inpatient rehabilitation in their clinical setting. The proportion of patients reported to be referred for inpatient rehabilitation ranged from 25% to 75%.

### Home-based PR referral

More than half of the pulmonologists who responded to a question on home-based PR (*n* = 47/83, 57%) referred 2–100% of eligible patients to a home-based PR program. The level of supervision required for home-based PR varied as follows: unsupervised (*n* = 17/47, 36%), supervised with a regular phone or video contact but no visits (*n* = 9/47, 19%), and supervised by a healthcare provider in the home for every session (*n* = 15/47, 32%). Most of the home-based rehabilitation programs lasted for 15–30 min (*n* = 24/4, 51%).

## Discussion

This study aimed to identify the facilitators of and barriers to referral to PR among pulmonologists in India. The validated questionnaire included domains related to pulmonologists’ perspectives and perceived patient parameters, exploring factors that either facilitate or hinder referrals to PR programs. While frequency analysis offers insight into the most reported barriers and facilitators, it is important to acknowledge that the most frequently reported reasons may not necessarily be the most significant or impactful factors influencing PR referral. The results are discussed under the following headings:

### Pulmonologists’ facilitators and barriers to referral to the PR program

Eighty percent of the pulmonologists reported that the limited number of centers offering PR programs and poor infrastructure may be a unique problem faced by developing countries. The reason is that the healthcare delivery system often permits low priority for setting up rehabilitation clinics over conventional outpatient clinics and emergency and intensive care units, as reported previously by Bansal V et al.^20^ The PR model of care practiced in an Indian healthcare system emphasizes acute management rather than proactive care for chronic conditions. Moreover, in a developing country such as India, the healthcare system is run by public and private facilities that include modern and complementary medicine, such as Yoga, Unani, Ayurveda, and homeopathy^21^. PR programme conducted in the private sector lack the necessary infrastructure, such as adequate space, equipment and trained professionals^14^. Furthermore, programs do not include the recommended core components^22^, and referrals to them are infrequent. Furthermore, there are no rehabilitation facilities in government hospitals for individuals with chronic disabling respiratory conditions^13^.

Thirty eight percent of the pulmonologists reported that the barriers faced in accomplishing the minimal attendance to the PR program (biweekly supervised sessions for eight to twelve weeks) were the distance of the rehabilitation centre from home, travel, and transport. Similarly, if the PR centre was in close proximity, it was considered a facilitator. This finding is consistent with a previous study reporting that the acceptance of a PR referral by patients is less likely as the distance from home to the rehabilitation centre increases^23^. Moreover, Sahashrabudhe. S et al. highlighted transportation as a significant barrier, with 58% of participants reporting reliance on public buses and many facing barriers related to long travel distances and inadequate infrastructure^24^. Patients without personal or family transport must depend on public transport (unfortunately, most modes of public transport are not adapted for patients with chronic respiratory illnesses), making it challenging; as a result, they often rely on taxis, which further adds to their healthcare expenses. Perhaps the inconvenience of accessing the rehabilitation centre coupled with a lack of transportation might prevent the treating pulmonologists from referring patients to the PR program. It is not clear whether these practical challenges prevent pulmonologists from discussing PRs altogether or if they still encourage participation despite these barriers. This topic deserves to be explored further.

The healthcare burden of chronic respiratory diseases results in significant healthcare costs. In our study, we found that the burden of increased healthcare costs was another factor that results in low referral to a PR program, as reported by 70% of the pulmonologists. In India, the direct medical average total cost of hospitalization and medications for patients with COPD during every visit was Rupees 29,885, which is approximately 300 Pounds^25^. Additionally, indirect caregiver costs in India accounted for 1,544 Rupees, which is approximately 18–20 Pounds^26^. Furthermore, in developing countries, the cost of rehabilitation is at the expense of the patient, which increases the individual’s healthcare cost. Advocation of the potential health benefits and cost-effectiveness of PR by healthcare professionals and patients may help increase payers’ awareness and knowledge of PR. This would further accelerate the implementation and delivery of PR^27^.

Furthermore, our results indicated that the treating pulmonologists’ belief in PR benefits has been shown to facilitate referral, a finding that is consistent with a previous study that reported that the perceived knowledge and understanding of the health benefits of PR by treating doctors is essential for accelerating the uptake of the PR program^28^.

In our study, patients’ level of physical disability was another factor perceived by pulmonologists to facilitate PR referral. Patients with both high and low levels of physical disability may be referred to PR programs by pulmonologists. Watson JS et al. reported that patients with a high level of physical disability (modified medical research council dyspnoea score of 3, 4, or 5) were more often referred^29^. However, our questionnaire failed to identify at which level of physical disability the pulmonologists referred their patients to the PR program.

Other factors, such as the patient’s level of motivation, comorbidities, and long-term oxygen therapy, were reported by pulmonologists to facilitate PR referral. Keating A et al. reported that patients who perceived greater benefits from PR, reflecting higher motivational readiness, were more likely to attend and complete the PR program^30^. In our study, we found that comorbidities or restricted activities influenced referrals to a PR program. As the PR program helps patients move around, make them functionally independent via assistive devices, and promote self-management strategies, pulmonologists might feel compelled to refer patients with comorbidities or restrict activities to the PR program.

### Pulmonologists’ perceptions of patient facilitators and barriers

This study aimed to identify patient-related factors that prevent and/or promote patient enrolment in PR programmes from pulmonologists, as pulmonologists are primary health care professionals who refer patients to PR programs. We assumed that patients often reported their reasons or willingness to attend the PR program to the treating pulmonologists. For example, in categorizing the identified barriers, we intentionally included both direct responses from pulmonologists and their perceptions of patient-level barriers. To this end, the item “financial constraints for patients” was classified under pulmonologist factors, as it reflects the pulmonologists’ clinical judgment and direct experiences—such as refraining from recommending certain interventions owing to anticipated financial hardship on the patient’s part. Conversely, “lack of financial assistance” was placed under pulmonologists’ perceived patient factors, representing how pulmonologists interpret or report patients’ expressed concerns about the absence of financial support systems (e.g., insurance coverage, government schemes). This dual approach allows us to capture both the professional decision-making context and the perceived socioeconomic realities of patients, as observed by healthcare providers.

In our survey, pulmonologists perceived that a lack of awareness of PR benefits was a common factor cited by the patients who refused to participate in PR, which resonates with the findings of Thakrar R et al., who reported that the level of awareness of PR was only 25.14%^31^. This may be addressed by conducting community campaigns and education^32^ to disseminate the health benefits of PR and help optimize its utilization^33–35^. Travel and transport were found to be another perceived barrier by pulmonologists when a patient refused a PR referral. Previous authors reported that patients whose house was at a distance of more than 36 miles from the centre or who needed to travel more than 30 min were less likely to complete the programme because they lacked financial assistance^36,37^. Although our survey did not specifically assess the impact of air pollution, it is important to consider that in areas with high levels of pollution, outdoor activities may worsen respiratory symptoms and could act as an additional barrier to accessing PR for individuals with chronic respiratory diseases.

The implementation of alternate models of PR delivery, such as home-based rehabilitation^38,39^ and telerehabilitation, may improve access to PR programs^40^. However, in low- and middle-income countries, the feasibility of telerehabilitation can be limited by costs related to devices, internet access, and digital literacy^14^. Additionally, not all patients are suitable candidates for telerehabilitation because of their clinical condition, technological barriers, or lack of support at home^14^. Additionally, the development of healthcare programs in high-burden settings, supported by adequate investment, may help address barriers to rehabilitation access^26^. The National Institute for Health and Care Research (NIHR) Global Health Research Group on Respiratory Rehabilitation (Global RECHARGE group) brings together collaborators from the UK, Kyrgyzstan, India, Sri Lanka, and Uganda to develop effective PR programs, highlighting the global need to tailor these initiatives to diverse healthcare settings^41^. Additionally, a partnership between the NIHR-funded Global RECHARGE group and the International Primary Care Research Group has delivered a ‘Teach the Teacher’ program to build PR capacity, adapting clinical and educational content for partners in India, Sri Lanka, Kyrgyzstan, and Uganda^24,42–46^.

In contrast, facilitators for the patient, as perceived by the pulmonologists, were identified. Good support provided by family members to patients during illness with coping strategies would favour the acceptance of PR referrals. This may further improve patients’ level of motivation; for example, the feelings of being isolated and depressed are curtailed through good family support. The easy accessibility of the rehabilitation centre was found to be another facilitator. Watson JS et al. reported that locating a centre close to the patient’s residence or consulting hospital might enhance the referral to the PR program^29^.

The positive influence of pulmonologists , patients’ positive beliefs toward rehabilitation and good health literacy were reported as facilitators in our study. This finding is consistent with Keating et al., who suggested that patient participation in PR is strongly influenced by clinician recommendation and referral strength^30^.

While evidence on the benefits of PR in developing countries is limited, several studies highlight common barriers such as a lack of awareness among physicians, resource shortages, and patient-related issues such as distance, financial hardship, and behavioural factors^43–45^. Healthcare workers in Uganda and Kyrgyzstan reported that patient comorbidities, social circumstances, and stigma further hinder participation^43,46^. Systemic barriers include a shortage of trained professionals, inadequate infrastructure, and financial constraints^47^. Interestingly, COVID-19 has facilitated the adoption of tele-rehabilitation as an alternative^47^. Other facilitators identified include community-based delivery, local sourcing of equipment, patient motivation, and increased public awareness, suggesting that tailored, resource-sensitive strategies are essential to improve PR access in these settings^47^.

Caution is required in interpreting the findings and generalizing, as the response rate to the survey was low and response bias cannot be ruled out. The use of a survey or questionnaire to explore barriers to and facilitators of PR referrals inherently limits participants to a set of predetermined response options defined by the researchers. This structured format may restrict the ability to capture unanticipated factors or nuanced insights that could emerge through more open-ended or qualitative methods. As a result, important contextual or emerging themes relevant to PR referral practices may remain unidentified, limiting the depth and scope of the findings. In the survey, a list of facilitators for PR referrals were provided, and “level of physical disability” was one of the options. This list was ambiguous, as it did not highlight whether a higher level of physical disability was the facilitator or the lower level of physical disability. Many of the questions in this study rely on pulmonologists’ recall, which introduces the potential for recall bias. Pulmonologists may overestimate or underestimate the proportion of patients referred for PR. Such inaccuracies can affect the validity of the findings by either inflating or deflating referral rates.

## Conclusion

Our study identified various facilitators and barriers faced by pulmonologists for PR referral, along with patient facilitators and barriers from the pulmonologists’ point of view. The key facilitators included the proximity of the PR centres and the availability of trained professionals, whereas the major barriers were financial constraints and limited programme availability. These findings can guide the development of tailored referral pathways in resource-limited settings to improve PR access and uptake. Future research should investigate the underlying reasons why patients choose not to enrol in PR to better understand and address barriers to engagement in these programs. .

## Data Availability

The data are available from the corresponding author. The authors declare that the study data are available upon reasonable request.
